# Successfully saving a child with destuctive iliac artery damage by hybrid surgery

**DOI:** 10.1186/s13019-024-02851-3

**Published:** 2024-07-27

**Authors:** Jianfeng Chen, Wei Zheng, Tingting Liu, Xianling Li, Zhong Xin, Zhonglong Han, Yingfeng Wu

**Affiliations:** 1 Department of Vascular Surgery, XuanWu Hospital, Capital Medical University, 45 Changchun Street, Xicheng District, Beijing, 100053 China; 2 Beijing Children’s Hospital, Capital Medical University, National Center for Children’s Health, Beijing, China

**Keywords:** Iliac artery injury, Vascular grafts, Interventional therapy, Pediatric trauma

## Abstract

Non-iatrogenic trauma of the iliac artery is rarely reported but is always life-threatening. In this report, we describe the case of a child with complete transection and partial disappearance of the iliac artery caused by bicycle handlebar impalement. He experienced catastrophic hemorrhage, malignant arrhythmia, and difficulty in exploring transected vessel stumps. Aggressive infusion, blood transfusion in time, and pediatric vascular characteristics help delay the deterioration during anesthesia induction. Eventually he was successfully rescued by performing interventional balloon occlusion and open revascularization after more than 7 h post-trauma. A series of interventions and precautionary methods may benefit such severely injured patients; thus, these methods should be highlighted.

## Introduction

Traumatic iliac artery injury rarely occurs, but it is known to be life-threatening. Its incidence has been reported to be as low as 0.2–0.4% amongst all pediatric traumas [1, 2], whereas the mortality rate due to pediatric iliac artery injury may reach as high as 72% [1, 3–5]. Complete transection with partial disappearance of the iliac artery — a serious infrequent situation, may be one of the most serious vascular injury types, because it can inevitably cause catastrophic hemorrhage and life-threatening extremity ischemia in a short time period. Here, we report the case of a patient who sustained traumatic impalement from a bicycle handlebar to the right groin that resulted in catastrophic hemorrhage, malignant arrhythmia and difficulty in seeking transected vascular stumps. Eventually, he was successfully rescued. The rare occurrence and lack of familiarity with this injury, combined with several life-threatening conditions included malignant arrhythmias, shock, ischemia–reperfusion injury,etc. make this case crucial; thus, it needs to be highlighted.

## Case Report

**Fig. 1:**
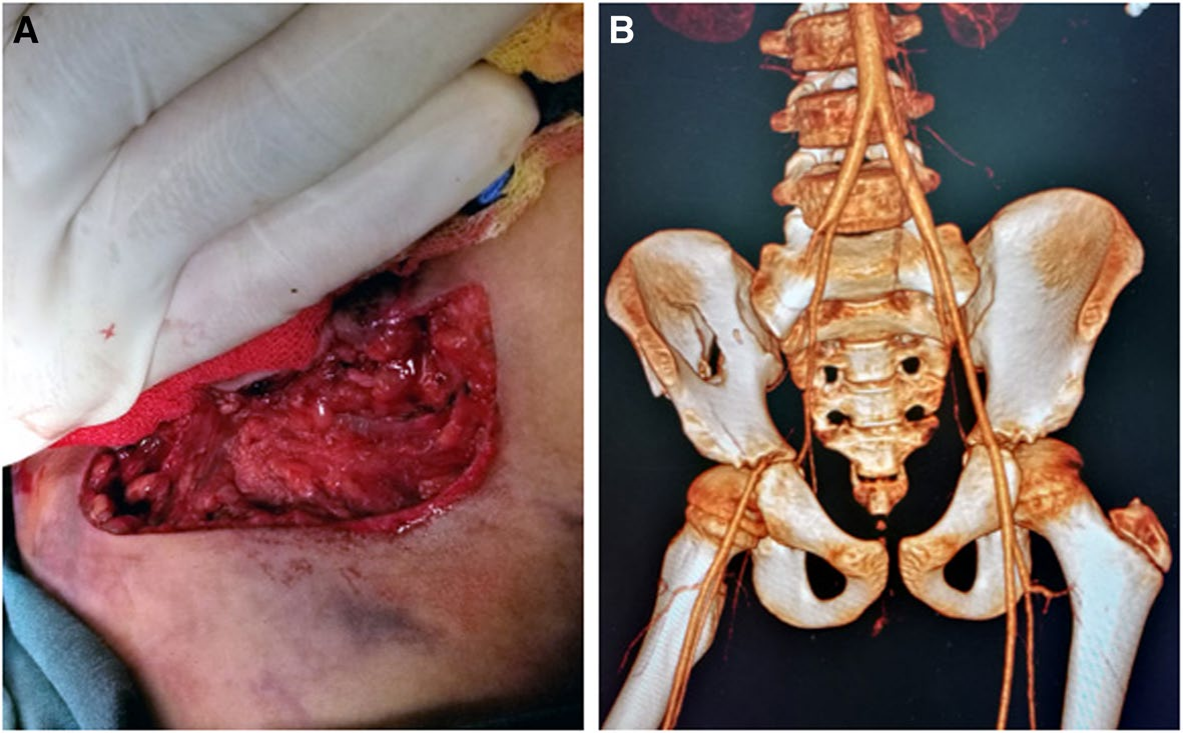
**A** An irregular wound approximately 12 cm in length in the right groin, which is under external manual compression. **B** CT scan detected fracture of right ilium wing, EIA was tapered to be completely transected. The common femoral artery was redeveloped through branches of internal iliac artery

A 10-year-old boy weighing approximately 35 kg was transferred to the emergency room of the National Center for Children’s Health, complaining of right lower abdomen handlebar impalement with bleeding for more than 7 h. He accidentally fell down while riding a bicycle that afternoon, and the bicycle metal handlebar was inserted obliquely from the right groin into the abdominal cavity. After pulling out the handlebar by himself, the blood spurted out of the wound and he fell and lost consciousness. He was transported to two hospitals in the last 7 h and received 2 units of blood. Upon arrival, his pulse was 78 beats per min, respiratory rate, 22 breaths per min, and blood pressure 99/50 mmHg, GCS score of 9. The patient is mildly impaired in consciousness and has an apathetic expression, pale with abdominal distension, and with blood oozing under continuous external manual compression. Blood was continuously gushing out of an irregular wound approximately 12 cm in length once the compression was removed (Fig. 1A),Initial examination of the wound revealed an obliquely oriented, deep wound with contusion of the surrounding tissues, accompanied by a large local hematoma and uncontrollable active bleeding. His right lower limb was externally abduction, with a lower skin temperature, weaker pulsation of the dorsalis pedal and posterior tibial arteries, and limited autonomous activity. Laboratory tests identified hgb of 59 g/L; wbc, 19.52 × 10^9^/L; HCT, 17.0%; Ca, 1.43 mmol/L (normal reference range, 2–2.75); amylase, 360 (0–125) U/L; prothrombin time, 19.9 (9.4–12.5)s; and fibrinogen, 0.76 (2.00–4.00) g/L. Computed tomography (CT) detected a fracture of the right ilium wing. The right external iliac artery (EIA) was tapered to be completely transected caudally from its origin. The right common femoral artery was supplied by the abnormal branches of the right internal iliac artery (Fig. 1 B).

**Fig. 2:**
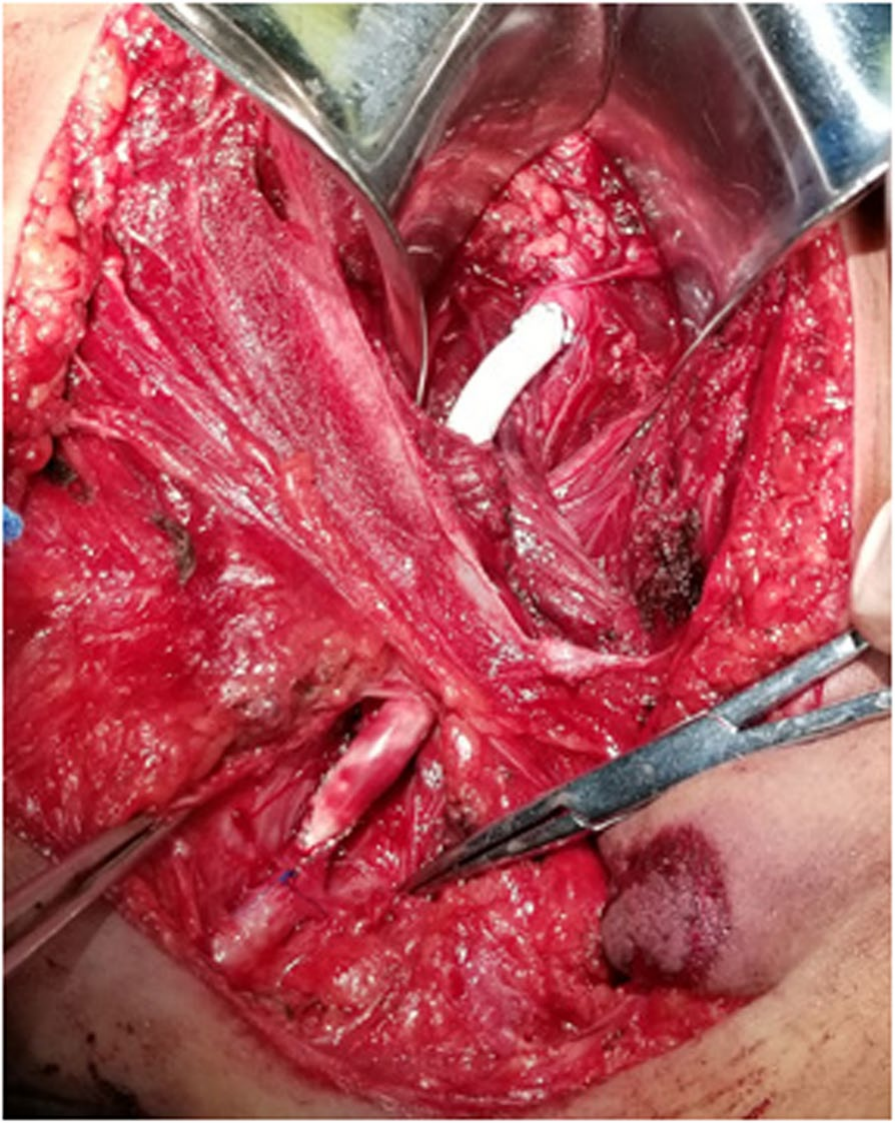
An proximal end-to-end anastomosis using a 4-mm-diameter Dacron artificial vessel and an distal end-toside anastomosis with common femoral artery

**Fig. 3:**
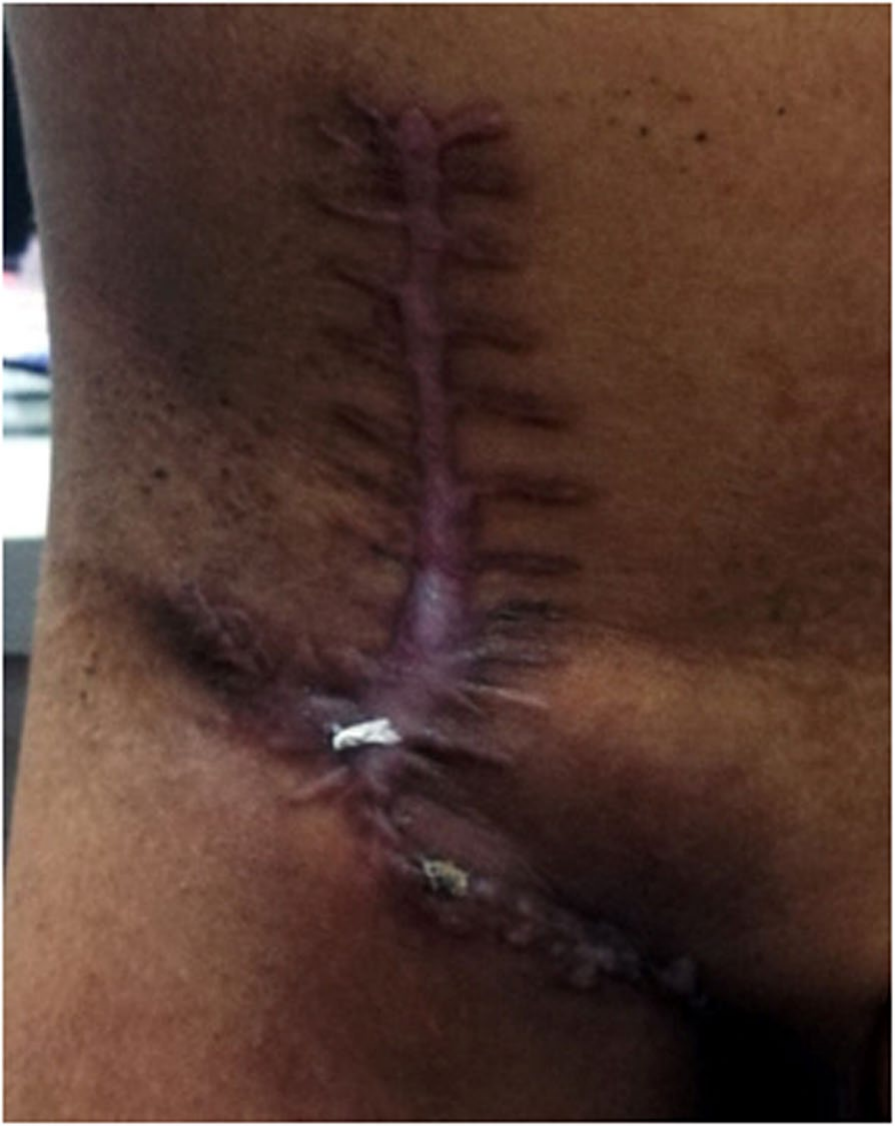
The healed wound

We immediately established multiple intravenous accesses for rapid fluid resuscitation and performed emergency transfusion protocols. Meanwhile, the emergency green path was opened, the child was transferred to the operating room. During the induction of anesthesia, the patient suffered from malignant arrhythmia (which means heart rhythm disturbances that can cause a life threatening emergency such as sudden cardiac arrest), and defibrillation was performed simultaneously with pressurized fluid infusion and blood transfusion, soon the sinus rhythm was recovered. Upon stabilizing the anesthesia, a laparotomy via the original traumatic wound was performed. The injured EIA terminal was found to be not constricted within where it should be, but moved obliquely to somewhere deeper in the abdominal cavity. After loosening the exterior compression, active bleeding was still conspicuous, still the bleeding site could not be found. Therefore, the operation plan was changed to control the bleeding by endovascular intervention firstly. Therefore, a 5-F sheath was placed in the left femoral artery, through which a 5–80 mm balloon catheter (Invatec Medtronic, Baja California, Mexico) was placed in the right common iliac artery and the right EIA after turning over under the guidance of a 260 cm guidewire. After filling up the balloon, the active bleeding stopped. During laparotomy, severe tissue contusion was found, and the hematoma formed by bleeding infiltration in the retroperitoneal soft tissue changed the normal anatomical structure. After removal of the free blood clot, a trauma-induced peritoneal perforation with local mesenteric haematoma was identified. Fortunately, the rupture of intestinal canal and intra-abdominal hemorrhage were not found. After the ballooned tissue was touched and fixed through bare hand touch, the blocked balloon was released. The iliac vein was intact, and no dissection of the distal iliac artery was found Therefore, the proximal broken end of the EIA was incised, with an end-to-end anastomosis using a 4-mm-diameter Dacron artificial vessel. At the other end of the Dacron vessel, an end-to-side anastomosis with the right common femoral artery was performed (Fig. 2). During this procedure, the right common femoral artery was ensured to be freed as far as possible to the proximal and then sutured. The pulsation of the right dorsalis pedal and posterior tibial artery returned to normal. The postoperative disease course was uneventful. After a 15-day bedrest, the wound healed well (Fig. 3) and did not have post perfusion syndrome features or compartment syndrome manifestations in the right lower extremity. He was discharged from the hospital for further rehabilitation, regular outpatient reviews and telephone follow-ups.

## Discussion

Handlebar injury of the iliac artery is rarely reported in the literature. Levi et al. described a case of crushed iliac artery injury resulting in thrombosis, wherein relief of ischemia by thrombectomy [6]. AA Singla et al. highlighted a delayed presentation of EIA occlusion secondary to a handlebar blunt injury in a pediatric patient who was preformed thromboendarterectomy [7]. Ko et al. reported the case of a 33-year-old man who suffered from complete transection of the common iliac artery after blunt abdominal trauma and was saved by performing emergent abdominal aortic and right common iliac arterial anastomosis with a vascular graft [8]. However, in the literature review, we did not find a similar life-threatening blunt-penetrating injury report, including destruction and partial disappearance of the iliac artery, catastrophic hemorrhage, and peri-cardiac arrest during the anesthetic induction due to decompensated hypovolemic status, as mentioned above.

The reason the child did not go into rapid shock or even die from trauma or blood loss during nearly 10 h from injury to surgery can be attributed to the following.. First, no fatal injuries, such as internal organ ruptures, were found to be associated with the ilium wing fracture, as demonstrated by surgical exploration. Second, the loss of fluids was compensated by aggressive infusion and blood transfusion during both transfers, which sustained the vital signs. Third, continuous external pressure resulted in a significant reduction of blood loss. Fourth, the iliac artery, a muscular artery, without arteriosclerosis in a 10-year-old boy, under a violent blunt traction force to the point of vascular rupture site caused by the bicycle handlebar, elastically contracted and became tail-like ends on CT angiography. The tunica media retracted into the adventitia composed of loose connective tissue, causing the vessel lumen to shrink obviously, thus limited the aggressiveness of blood loss.

Cardiac arrest during anesthesia is also not accidental [9]. Although compensatory mechanisms such as transfusion and redistribution of body fluids are at work, anesthesia-induced vasodilation leads to a sudden relatively decreased blood circulation. As a result, severe shortage of oxygen supply to the heart lead to a malignant arrhythmia or even cardiac arrest. During the revival process, defibrillation cannot often maintain or even restore normal heart rhythm, whereas adequate rapid blood transfusion and appropriate vasoactive drug application may be more necessary. During nearly 6 h of resuscitation, Exactly 8 U of hematocrit and 800 mL of fresh plasma were infused.

The initial protocol was based on the premise that the vascular trauma area could be directly visualized for hemostasis and reconstruction. An effort towards searching the severed blood vessels would have aggravated the trauma and quickly led to excessive bleeding or cardiac arrest, which are extremely dangerous. In fact, the destroyed retroperitoneal anatomy, combined with retracted arteries and uncontrolled active bleeding often lead to a failure to find the EIA stumps. Changing the operational course to endovascular interventional balloon occlusion was proved correct when we could not find the bleeding vascular stump, which highlights the significance of the hybrid surgery.

The earliest reported use of balloon occlusion in trauma was during the Korean War [10]; however, it was used in the aorta and in adults. Prophylactic balloon occlusion of the internal iliac arteries was first described in 1997 [11] and has been widely accepted since then. In a recent review that discussed the trends in management of vascular trauma, Patel JA showed that the placement of a balloon in the infrarenal aorta may temporarily support resuscitation and provide time to prepare the operating room in hemodynamically unstable patients [12]. Unfortunately, we believe that this procedure undoubtedly results in contralateral limb ischemia and incomplete occlusion of the traumatized limb. In our case, successful occlusion at the severed stump of the EIA allows more leisure exploration and revascularization. We did not find such a report in our literature review.

Finally, the dilated balloon should be noted as an easy marker for locating the severed end of the EIA. As mentioned earlier, bruised soft tissues and retroperitoneal hematomas are known to have destroyed the normal anatomy, and the retracted severed artery had elastically retracted among the loose adventitia, makes it extremely difficult to isolate and locate the severed vessel stump under direct vision. With the touch of the fingers, it is very easy to reveal it. In fact, EIA was not identified until its middle layer was exposed after further trimming.

## Conclusion

We present a rare and severe case of iliac artery transection trauma caused by bicycle handlebar, and a way of successfully rescuing the patient through hybrid surgeries. When faced with a young patient with a ruptured iliac artery, early compression for hemostasis, fluid resuscitation, and surgical repair of the damaged vessel should be performed as soon as possible. Aggressive infusion and blood transfusion should be taken to prevent arrhythmia and cardiac arrest during anesthesia induction. The hybrid surgery of endovascular balloon occlusion followed by open repair and revascularization should be a priority.

## Data Availability

No datasets were generated or analysed during the current study.
